# CNNdel: Calling Structural Variations on Low Coverage Data Based on Convolutional Neural Networks

**DOI:** 10.1155/2017/6375059

**Published:** 2017-05-28

**Authors:** Jing Wang, Cheng Ling, Jingyang Gao

**Affiliations:** Department of Computer Science and Technology, Beijing University of Chemical Technology, Beijing, China

## Abstract

Many structural variations (SVs) detection methods have been proposed due to the popularization of next-generation sequencing (NGS). These SV calling methods use different SV-property-dependent features; however, they all suffer from poor accuracy when running on low coverage sequences. The union of results from these tools achieves fairly high sensitivity but still produces low accuracy on low coverage sequence data. That is, these methods contain many false positives. In this paper, we present CNNdel, an approach for calling deletions from paired-end reads. CNNdel gathers SV candidates reported by multiple tools and then extracts features from aligned BAM files at the positions of candidates. With labeled feature-expressed candidates as a training set, CNNdel trains convolutional neural networks (CNNs) to distinguish true unlabeled candidates from false ones. Results show that CNNdel works well with NGS reads from 26 low coverage genomes of the 1000 Genomes Project. The paper demonstrates that convolutional neural networks can automatically assign the priority of SV features and reduce the false positives efficaciously.

## 1. Introduction 

Genomic structural variation, usually longer than 50 bp [1], is one of the most important types of genetic mutations, which potentially leads to severe diseases, cancers, and even death by breaking the structure of chromosomes. For example, the deletions in ADAM17 are linked to inflammatory skin and bowel diseases [2]. Lee et al. have shown that a variety of prenatally diagnosed congenital heart diseases are related to 22q11.2 deletions [3].

NGS [4] parallelizes the sequencing process and produces massive short reads within 400 bp, which are aligned to the reference sequence by reads mappers like Burrows-Wheeler Aligner (BWA) [5] and Bowtie2 [6]. The alignments of reads are often stored in SAM or BAM format devised by SAMtools [7]. The data mapping step filters anomalously mapped reads, which are direct evidence of SVs.

Most existing SV callers are classified into four categories [8]: (1) discordantly mapping read pairs (i.e., two reads in a pair cross the SV region, and the distance between them is inconsistent with the insert size); (2) split reads: split reads are subdivided into soft-clip reads (i.e., one of the paired reads is partially mapped) and one-end-anchored reads (i.e., one of the paired reads is unmapped); (3) read depth (i.e., the number of reads covering a region); (4) local contig assembly (i.e., assemble reads to form longer consensus sequences, which are called contigs, and then remap them to the reference genome). Many NGS-based SV detection methods have been proposed based on these four theories. These SV detection methods vary in both accuracy and sensitivity, since they utilize different properties to assess the likelihood of SVs.

Each method has its own advantages on the judgement standards of SVs. Take deletion as an example, which is the most common mutation in structural variation [9]. Pindel [10] concentrates on one-end-anchored reads. It performs poorly under low coverage. BreakDancer [11] compares insert size and the separation distance between discordant paired reads to ascertain breakpoints. SVseq2 [12] and DELLY [13] are hybrid approaches to call SVs. SVseq2 applies an enhanced split-reads mapping algorithm to identify deletions and filters the candidates with discordant read analyses. DELLY, on the contrary, uses discordant reads to find candidate SVs and then verifies the exact breakpoints by split-reads alignments. Unsatisfactorily, all these tools produce low accuracy and sensitivity on low coverage sequence datasets. MetaSV [14], a recently proposed method, combines the results derived from many direct SV calling tools and verifies the candidates using local assembly to reduce false positives rate. Such integrated SV callers still suffer from low accuracy despite relatively high sensitivity. It is worth learning that MetaSV places higher weight to more accurate split-reads methods than discordant paired reads methods.

In this paper, we introduce a SV caller named CNNdel. CNNdel utilizes a convolutional neural network model to accomplish the false positives filter procedure. Compared with other integrated methods, CNNdel is capable of automatically assigning the weights of SV features by neuron networks and the detection accuracy on low coverage real data greatly outstrips the prior methods.

## 2. Background

Convolutional neural network (CNN) [15] is a typical supervised deep learning algorithm, which is widely applied in image and video recognition, such as face recognition, license plate recognition, and motion prediction in video. For example, the famous LeNet-5 network is applied to recognize handwritten characters [16].

CNN consists of multiple convolutional layers and pooling layers, following full connected networks as hidden layers and the output layer. Each neuron in convolved layers is connected to a small region of the previous layer. Convolution operation is executed with the input of the small region and a filter. The products are summed up as the value of the current neuron. Each convolved layer contains a set of feature maps. Each map has its own filter or kernel. Pooling is a form of nonlinear downsampling. For example, in max-pooling, the input matrix can be divided into nonoverlapping small regions, and for each small region, the layer outputs the maximum. Similarly, in average-pooling or mean-pooling, the layer outputs the average values of each small region.

There are many popular deep learning software frameworks such as Caffe [17], Theano [18], TensorFlow [19], and Torch [20]. The paper [21] gives a detailed presentation about these frameworks. The latest neural network library Keras [22] has attracted wide attention. Taking Theano or TensorFlow as backend, Keras models minimalist and highly modular networks. Other than frameworks that support many kinds of deep architectures, Keras is designed for convolutional networks and recurrent networks. In this paper, Keras is chosen to model a CNN classifier. To further confirm the performance of CNNdel, parameters of the CNN classifier are regulated.

## 3. Method

**Figure 1:**
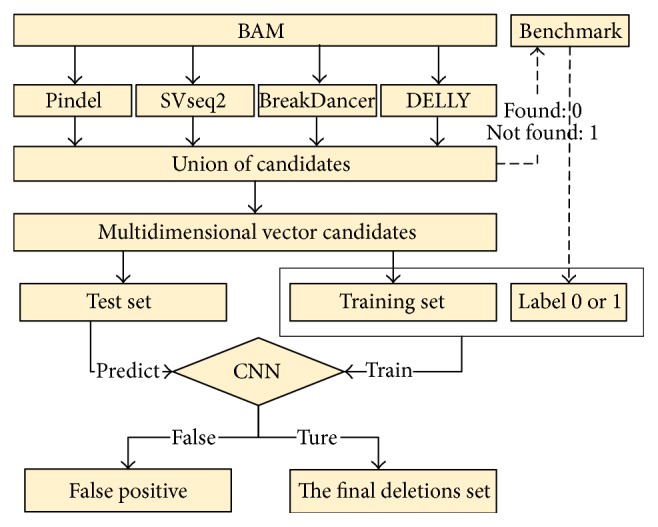
CNNdel pipeline. The pipeline is generalized to 4 steps: (1) get the union of candidates resulting from four tools; (2) get the feature information of each candidate; (3) label each candidate; and (4) use labeled candidates to train the CNNs and validate the trained model.

In this paper, we focus on the calling of deletions. CNNdel is not a direct SV caller like Pindel or SVseq2; it collects the results from other tools. The pipeline of CNNdel can be generalized to a 4-stage process: (1) get the union of candidates derived from four prior tools by merging duplications; (2) extract features of each candidate; (3) label each candidate by checking the SV benchmark file; and (4) supervised by the labels, use a major part of candidates to train the CNN model and validate the trained model with the remaining candidates.  Figure 1 illustrates the framework of CNNdel.

### 3.1. Get the Union of Candidates

In order to get as many candidates as possible, Pindel, SVseq2, BreakDancer, and DELLY are run with default parameters. When the distance between two deletions is less than 2% of the shorter deletion length, they are considered as duplications. Learning from MetaSV, when a candidate is assigned with different bounds in the merging process, the bounds given by split-reads methods are more trustworthy.

### 3.2. Check the Feature Information of Candidates

By checking the features which distinguish deletions from the normal sequence regions, we transform each candidate deletion into a multi-dimensional vector. Five major feature types are specified as:

- Feature (1) (deletion length): split-reads mapping reacts badly on overlong deletions. Longer deletions are likely to have different reads distributions with shorter deletions. It is essential to add the length of a deletion as a feature.
- Feature (2~9) (consistency of mapped read pairs): discordant mapped read pair is one of the most direct lines of evidence to support the existence of a deletion. For the discordant and concordant mapped read pairs, refinement works are demanded. Both are, respectively, subdivided into two branches: (i) read mapping error (i.e., note whether the mapped reads are error-free or with mismatches, since the reads mapper BWA is designed to allow mismatches) and (ii) read mapping uniqueness (i.e., note whether a read is uniquely mapped or can be mapped to multiple positions).
- Feature (10~33) (split-reads analysis): the reads overlapping the breakpoints of the deletions can be classified into three sorts: (i) fully mapped, (ii) soft-clip (the read cannot be mapped as a whole but its prefix or suffix part can be mapped), and (iii) one-end-anchored (one read in a pair can be mapped while the other one is unmapped). These three sorts are, respectively, subdivided into three detailed branches: (iv) breakpoint positions (the reads overlap whether with the left or the right breakpoint), (v) anchor positions (the mapped one in a pair lies whether upstream or downstream of the deletion region), and (vi) reads mapping uniqueness.
- Feature (34~37) (read depth): the depth in deletion regions is close to 0, since few reads can be mapped to the region. Use SAMtools to count the depths of reads within the deletion region, reads upstream of the deletion region, and reads downstream of the deletion region. The depth of a region is defined as ∑_*i*=1_^*l*^depth(*i*)/*l*, in which depth(*i*) is the depth of the *i*th base in the region and “*l*” is the length of the region. The three counts are normalized to four values between 0 and 1 in preprocessing.
- Feature (38~49) (mapping reads statistics): the last feature type counts the depths in and around the deletions. In this type, the eligible reads in the same three regions are counted: (i) reads within the deletion region, (ii) reads upstream of the deletion region, and (iii) reads downstream of the deletion region. These reads are sorted by (iv) reads mapping error and (v) reads mapping uniqueness.

All features are listed in Table 1. Searching in and around a candidate region according to its known chromosome ID, individual ID, and start and end positions, reads which match the above conditions are counted. Before being imported into the CNN model, these 49 features are normalized into decimals between 0 and 1 in preprocessing.

In the application of CNNs on images, the local receptive fields (sliding windows) are geographically relevant to the neighboring fields. CNNs training could fail when shuffling the pixels in images. As shown in Table 1, 49 features are initially ranked according to the five types. In the Results and Discussion, we will explore the impact of the order of features on the performance of CNNdel.

### 3.3. Label Each Candidate

Search the deletion benchmark files to inspect whether a candidate deletion is in it. Once a deletion is confirmed, it will be labeled as 1 or 0 otherwise. Thereby, the procedure of false positives filtering can be regarded as a supervised binary-classification problem.

### 3.4. Use Labeled Candidates to Train the CNNs and Validate the Trained Model

**Figure 2:**
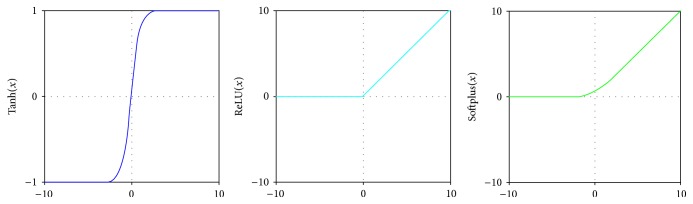
Typical activation functions: Tanh, ReLU, and Softplus.

(i) Layer structure: the convolved layer is abbreviated to “C.” The pooling layer is abbreviated to “P.” The networks' structure is usually set as “C1 + P1 + C2 + P2 + ⋯” following flattening hidden layers and an output layer.
(ii) Parameters: the convolutional neural network model is trained in a supervised way, and we optimize the weights of networks by stochastic gradient descent (SGD), which is given as
  (1) $$\begin{array}{c} \theta =\theta -\alpha \nabla_{\theta }J\left(\;\theta ;x^{\left(i\right)};y^{\left(i\right)}\;\right). \end{array}$$
“*θ*” is the weights of input features. “*α*” is the learning rate. The gradient ∇_*θ*_*J* gives the descent direction of weights. In gradient descent, all samples are calculated to decide the gradient, which costs massive time. To solve the problem, SGD learns the gradient on a batch-size number of samples, followed by a next round on other batch-size samples, until all samples run out. This procedure is called one epoch. Grid search method [23] is used to adjust the learning rate and batch. Smaller epoch prevents the classifying quality of CNN, while larger epoch has the risk of overfitting the model. Split a fraction of the training data as a validation set. Train only on the training set and monitor the validation error every few epochs. Early-stop method stops training as soon as the error on the validation set is higher than it was the last time it was checked.
(iii) Activation functions: activation functions are crucial factors in CNNs which bring about nonlinearity into networks.  Figure 2 shows the typical activation functions. Hyperbolic tangent (Tanh) function squashes a real-valued number to the range [−1,1]. It can be computed as Tanh(*x*) = (*e*^*x*^ − *e*^−*x*^)/(*e*^*x*^ + *e*^−*x*^). Rectified linear unit (ReLU), defined as ReLU(*x*) = max⁡(0, *x*), involves simple operations and accelerates the convergence of SGD compared to Tanh. However, ReLU sometimes frustrates the training model, since ReLU could prevent activating a neuron on data again in the weights updating procedure. Softplus function, a smooth approximation of ReLU, has the mathematical form Softplus(*x*) = log⁡(1 + *e*^*x*^). These rectifiers are called biological activation functions.
(iv) Input: CNNs are frequently applied in image recognition systems, in which the inputs are 2D images. Our samples are 1D text data. Thus, we regard our 1D data as 2D “images.” Each 49-feature deletion can be viewed as an image with 1 by 49 “pixels.” According to simple cross-validation [24], we randomly split all candidates into a training set and a test set. We use the labeled training set to train the CNN model and validate the trained model with the test set. The size of the training set is two times the test set.

## 4. Results and Discussion

Throughout the following experiments, we first recommend the befitting CNN model by adjusting different parameter settings. Secondly, it is substantiated that the order of 49 features has no crash to the final performance, but shuffling the order of training candidates generates adverse effects instead. Finally, the comparisons between CNNdel and the prior tools and comparisons between CNNdel and SVM are exhibited. Taking both accuracy and sensitivity into account, the parameter *F*-score is used to evaluate the performance of the CNN model. *F*-score is specified as “2 × accuracy × sensitivity/(accuracy + sensitivity).”

### 4.1. Experimental Environment and Dataset

Pindel, SVseq2, BreakDancer, DELLY, and CNNdel are implemented on an Intel(R) Xeon(R) CPU E5-1620 v2 @3.70 GHz, 16 GB RAM, and 1 TB storage with average disk access speed of 164.8 MB/s. Keras runs on Python 2.7 with the backend of Theano.

The raw sequences for the experiments contain 26 samples derived from chromosome 11 and chromosome 20 from human reference hs37d5. All reads are mapped to reference sequences by mapper BWA with default parameters, with BAM files as outputs. And the BAM files are indexed by SAMtools. Benchmark files are released by 1000 Genomes Project Phase III [25]. The mean insert size and mean read length are 425 bp (range: 237–579 bp) and 79 bp, respectively. As a low coverage dataset, the average depth covers 10.6x.

**Figure 3:**
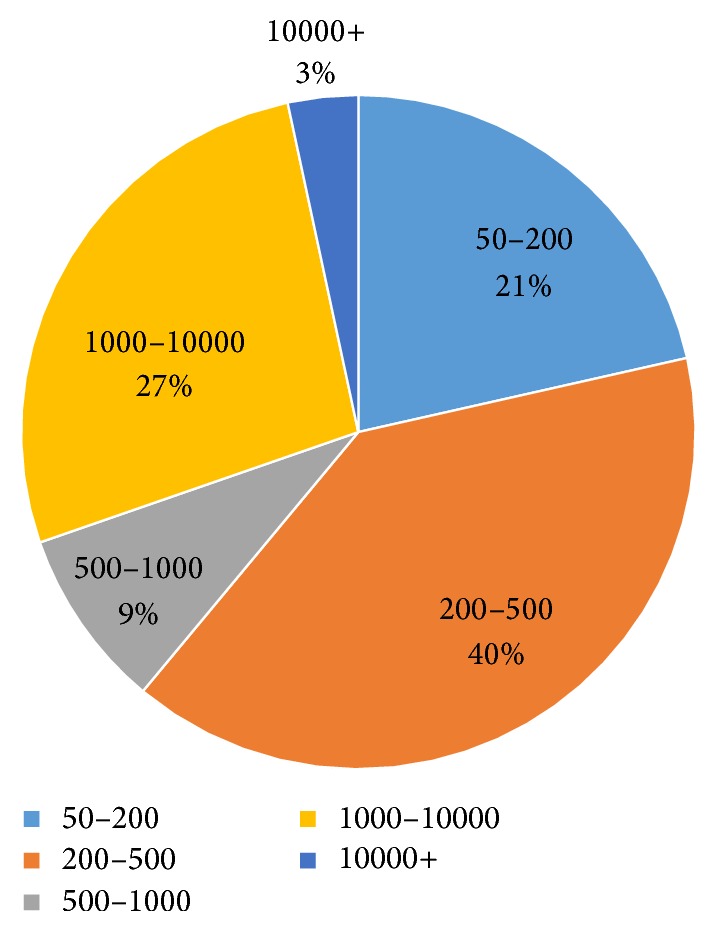
Length distribution of deletion datasets.

Figure 3 shows the length distribution of deletion datasets. There are a total of 2138 deletions in 26 samples. Copy number variations (CNVs) [26], defined as insertions or deletions that extend to 1 kilobase (kb) or above, occupy about 30% of total SVs. Medium-length deletions take the biggest share.

### 4.2. CNN Model Adjustment

#### 4.2.1. Layer Structure

Table 2 records the efficiency and run time of different structures. Learning rate and batch in the beginning are empirically initialized as 0.1 and 64. Tanh is employed as the initial activation function. The results show the following:

(a) The efficiency differs a little in the three kinds of structures.

(b) Structures with fewer layers spend less run time.

The simplest structure “C1 + P1 + F1 + F2” performs best whether in efficiency or in run time. However, fewer layers are accompanied by more weights, which increase the burden of memory.

#### 4.2.2. Learning Rate and Batch

In gradient descent or full batch learning, batch size is identical to the number of all training samples. Full batch learning and online learning (batch size is equal to 1) are two extreme situations. The number of training candidates is a little higher than 8000. So, in the beginning, the batch size is between 1 and 8192. As a general rule, learning rate is usually set as 0.1 and then divided by 2 or 5. Thus, learning rates are assigned as 0.1, 0.05, 0.03, 0.01, and 0.005. The initial activation functions are still set as Tanh.

**Figure 4:**
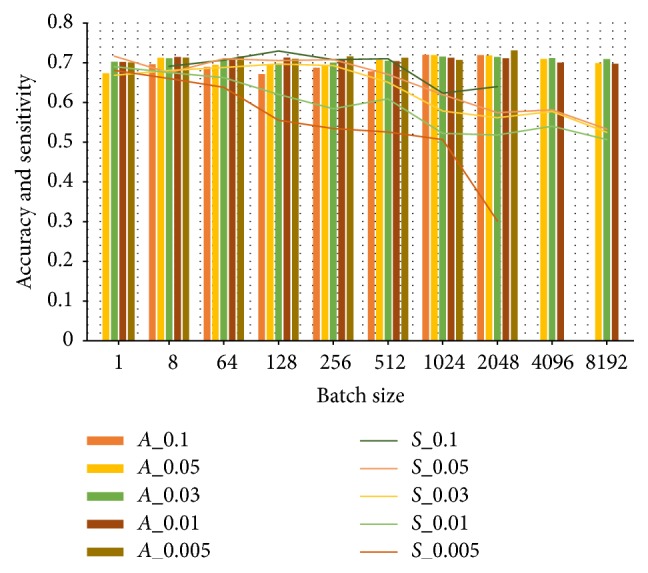
Grid search results of learning rate and batch. “*A*” in the legend means accuracy. “*S*” in the legend means sensitivity. The numbers after “*A*” and “*S*” are learning rates. The horizontal axis shows the batch range. The bar chart shows the accuracy, while the line chart shows the sensitivity.

Figure 4 illustrates the grid search results of learning rate and batch. The test results show the following:

(a) The accuracies of these learning rates keep stable around 0.7 when batch size varies.

(b) The sensitivities of learning rates descend with the increase of batch size.

(c) Larger learning rate outperforms smaller ones in sensitivity.

(d) Smaller learning rates always fail to train the model under a huge batch.

Besides, smaller learning rates make the model suffer longer running time. Thus, we conclude that 0.1 is the most appropriate learning rate. In such a case, Table 3 lists the performances of different batch sizes. Too small batch size (such as 1) hinders the convergence of networks, while too large batch size cuts down the times of iterations, leading to longer time to reach a good precision. Getting rid of less accurate batch values, the range 8–512 is appropriate whether in performance or in running time. It is suggested to assign 64 as the batch size since models achieve the highest *F*-score when batch is equal to 64.

#### 4.2.3. Activation Function

**Figure 5:**
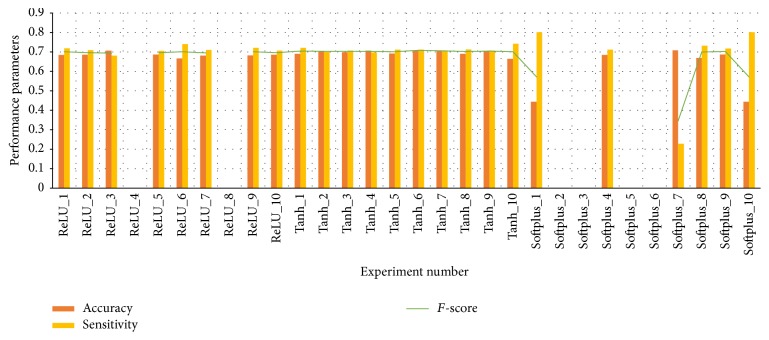
The performances of CNN models which are applied with Tanh, ReLU, and Softplus, respectively.

As shown in Figure 5, Tanh, ReLU, and Softplus are applied in CNNs in turn, with learning rate of 0.1 and batch assigned as 64. Each kind of model is run for 10 rounds to verify the stability of the model. The average run times are recorded as 10.2 s, 22.9 s, and 33.9 s when the models are applied with Tanh, ReLU, and Softplus, respectively. It can be concluded that, on accuracy and sensitivity, successfully trained models with Tanh and ReLU do not have significant differences. Softplus makes performance parameters abide violent fluctuation. Besides, models with ReLU and Softplus functions often die during training because they can prevent a neuron from being activated again. Thus, Tanh function stands out for its stability and decent performance.

Learning rate is confirmed as 0.1 for its speed advantage. To further confirm the reliability of the other recommended parameter settings, Table 4 displays the comparisons between combinations of layers, learning rate, activation functions, and batch sizes. The performances of the models are mainly influenced by activation functions. Networks employing Tanh functions always can achieve high accuracy and sensitivity with batch size in such a large scale (8–512).

Model mortality means the frequency with which a model fails during training. According to the highest *F*-score, the learning rate is suggested to be 0.1. It is advocated to use Tanh as activation functions and maintain “C1 + P1 + F1 + F2” as a hierarchical structure if the equipment can satisfy the memory requirements. As to the batch, we recommend smaller ones such as 8 and 64.

#### 4.2.4. Other Tricks about the CNN Model

Other tricks that are beneficial to the models are listed:

(a) Max-pooling or mean-pooling: mean-pooling costs longer time and receives similar results to max-pooling. We suggest using max-pooling in the model.

(b) Filter size and stride size: smaller filter (e.g., we use “1 × 4” as the filter size) and small strides (e.g., 1) help improve the accuracy of the CNN model.

(c) Regularization: dropout, a simple regularization technique, is applied to prevent overfitting. Dropout rate is tested from 0.5 to 0.1, and through testing 0.3 is suggested.

### 4.3. Shuffling

#### 4.3.1. Features Shuffling

**Figure 6:**
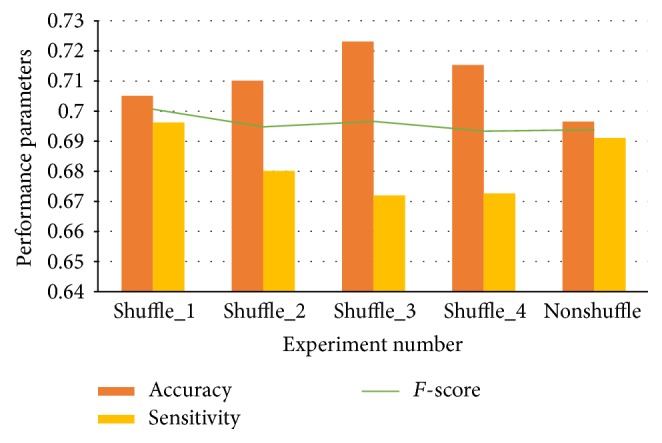
Impact of shuffling features.

With the experiments already shown above, the learning rate and batch are set as 0.1 and 64. 49 features are randomly shuffled. Results in Figure 6 certify that the accuracies and sensitivities of the successful trails have a little difference with the nonshuffled one.

Most detailed subclasses in the five main types are opposite, such as “mapped without error” and “mapped with mismatches” and “mapped uniquely” and “mapped to multiple positions.” Most features are biologically independent of each other. Thus, shuffle has a little effect on the efficiency of models.

#### 4.3.2. Candidates Shuffling

**Figure 7:**
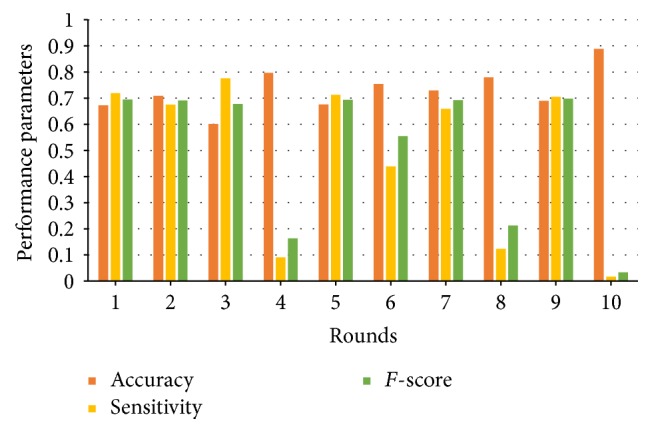
Impact of shuffling candidates.

It is hazardous to shuffle the 49-dimensional training candidates. Ten rounds of operating results shown in Figure 7 authenticate this conjecture. In such a case, the CNN model faces frequent frustrating results.

In the preprocessing stage, candidates derived from prior tools line up in the order of coordinates. Man-made translocations happen if two deletions switch their positions. There are features like “breakpoint positions” which are related to the relative positions. According to the experimental results shown in Figure 7, candidates without shuffle are recommended.

### 4.4. Comparisons with the Prior Tools

10 rounds of simple cross-validation are carried out to insure the reliability of CNNdel. In each round, gather the candidates of 9 individuals on chromosome 11 and chromosome 20 (70% of the candidates, totally 18 files) as the training set and the remaining as test set (30% of the candidates, 8 files). The average accuracy, sensitivity, and *F*-score of the 10 rounds are reported.  Table 5 compares the effectiveness of CNNdel with the prior tools. For Pindel, the parameters are set as “-w 0.1 -x 5.” SVseq2 is run with cutoff values 3. And other tools are run with default parameters in order to get as many candidates as possible.

With handcrafted features capturing reads distribution, CNNdel outperforms all prior methods in both accuracy and sensitivity. In comparison to the union results of the tools, CNNdel removes plenty of likely false positives and achieves a higher accuracy. However, it is possible for CNNdel to misjudge nondeletion candidates as deletion, which forces the emergence of false negatives. Thus, the sensitivity suffers a little decline compared to sensitivity of the union of tools. CNNdel largely preserves the sensitivity (mean loss of 8.4%) of the test set.

Table 6 denotes the accuracies of CNNdel and the prior tools in different deletion length ranges. SVseq2 outperforms the other tools for deletions in the length of 500 bp–1000 bp. BreakDancer and DELLY operate well on CNVs within the deletion length scope of 10000 bp. Despite a minute gap with SVseq2 on deletions of 500 bp–1000 bp, CNNdel outstrips these tools from a general view, especially on deletions longer than 10000 bp.

### 4.5. Comparisons with SVM

Both CNNs and SVM can perform well on false positive filtering with similar *F*-scores. The primary dissimilarity is that CNNdel exports stable performance all along while SVM deeply relies on the parameters and waves violently when the parameters are adjusted. Therefore, it requires a considerably long run time for SVM on grid search to adjust the parameters for the sake of better results.

In SVM, the penalty factor represents the tolerance to classification error. Radial basis function (RBF), one of the commonest kernel functions, is defined as function (2), in which *σ* stands for the width argument. Grid searches of SVM are carried out on the penalty factor (denoted by “*c*”) and the width argument (denoted by “*g*”):

(2) $$\begin{array}{c} k\left(\;\left\|\;x-x_{c}\;\right\|\;\right)=exp\left(\;-\frac{{\left\|x-x_{c}\right\|}^{2}}{\sigma^{2}}\;\right). \end{array}$$

Table 7 chooses the results of SVM when it performs best on accuracy, sensitivity, and *F*-score correspondingly.

CNN tends to outperform SVM significantly in accuracy when SVM receives better sensitivity. After repeated trials, SVM acquires a similar set of performance parameters.

## 5. Conclusion

In this paper, we propose a CNN-based method to call deletions on low coverage real data. CNNdel pipeline first collects the union of candidates derived from Pindel, BreakDancer, SVseq2, and DELLY and then finds all features of candidates. Afterwards, CNNdel searches the SV benchmark to get the labels of candidates. Finally, CNNdel trains the CNN model with labeled feature-presented candidates and filters the false positives out.

Based on the above experiments, in order to achieve better accuracy, CNNdel should be optimized by adjusting CNN model parameter settings, especially the activation functions. As a matter of fact, CNN model achieves stable accuracy and sensitivity when the structure, parameters, and activation functions vary in appropriate ranges. The impact of the order of features is also discussed. Experiments show that randomly shuffling the 49 features has a little influence on the performance of CNN. On the contrary, shuffling the order of training candidates causes severe damage to the results. The experimental results show that CNNdel outperforms other tools on low coverage real data. Not only CNN model, but also other nonlinear classification models such as SVM can remove the false positives, though it needs complex parameter regulations.

Efforts will be made to incorporate more strategies of SV detection to extract more cogent features. And CNNdel will be improved by modeling better deep learning networks. Besides, extensive experiments on genomes from patients will be conducted to realize higher clinical application value.

## Figures and Tables

**Table 1:** List of features to call deletions.

Feature types	Amounts
Deletion length	1
Consistency of mapped read pairs	8
Split reads analysis	24
Read depth	4
Mapping reads statistics	12

**Table 2:** Comparisons of different structures.

Layers	*A*	*S*	*F*	Run time
C1 + P1 + F1 + F2	0.7001	0.7081	0.704	23

C1 + P1 + C2 + P2 + F1 + F2	0.6894	0.7069	0.6980	30

C1 + P1 + C2 + P2 + C3 + P3 + F1 + F2	0.6845	0.7124	0.6981	54

“*F*” means *F*-score. The unit of run time is seconds. “*A*” indicates accuracy. “*S*” indicates sensitivity.

**Table 3:** Comparisons of different batch sizes with learning rate of 0.1.

Batch	*A*	*S*	*F*	Run time
1	0.6641	0.6574	0.6607	31
8	0.7024	0.6906	0.6964	20
64	0.7001	0.7081	**0.704**	30
128	0.6809	0.7213	0.7005	28
256	0.6907	0.7017	0.6962	24
512	0.6941	0.6915	0.6928	30
1124	0.7153	0.6237	0.6664	70
2048	0.7191	0.5988	0.6535	86
4096	0.7122	0.53	0.6077	107
8192	0.701	0.4817	0.571	113

“*F*” means *F*-score. The unit of run time is seconds. “*A*” indicates accuracy. “*S*” indicates sensitivity.

**Table 4:** Comparisons of different model parameters.

Layers	Activation	Learning rate	Batch range	Run time	Mortality	Space requirements
C1 + P1 + F1 + F2	Tanh	0.1	8~512	Medium	Hardly	Large
	ReLU	0.1	8~512	Fast	Medium	
	Softplus	0.1	1, 8, 64	Slow	High	

C1 + P1 + C2 + P2 + F1 + F2	Tanh	0.1	8~512	Medium	Hardly	Medium
	ReLU	0.1	8~512	Fast	Medium	
	Softplus	0.1	1, 8	Slow	High	

C1 + P1 + C2 + P2 + C3 + P3 + F1 + F2	Tanh	0.1	8~512	Medium	Hardly	Small
	ReLU	0.1	8, 64	Faster	Medium	
	Softplus	0.1	1, 8	Slow	High	

**Table 5:** Accuracy, sensitivity, and *F*-score of initial tools and CNNdel.

Tools	Accuracy	Sensitivity	*F*-score
Pindel	0.3957	0.5315	0.4537
SVseq2	0.5573	0.5813	0.5690
BreakDancer	0.3831	0.388	0.3855
DELLY	0.4064	0.448	0.4262
Union	0.4329	**0.7906**	0.5595
CNNdel	**0.6894**	0.7069	**0.698**

**Table 6:** Comparison with prior tools on different deletion lengths.

Deletion length	Deletions number	Pindel	SVseq2	BreakDancer	DELLY	CNNdel
50–200	147	0.2791	0.3233	0.2222	0	**0.53**
200–500	275	0.6484	0.6738	0.3347	0.3191	**0.8**
500–1000	65	0.7576	**0.8611**	0.4920	0.5658	0.72
1000–10000	172	0.575	0.6606	0.7203	0.7181	**0.88**
10000+	24	0.09	0.375	0.5	0.2857	**0.72**

**Table 7:** Accuracy, sensitivity, and *F*-score of initial tools and CNNdel.

Tools	Accuracy	Sensitivity	*F*-score
SVM (*c*: 1 *g*: 3)	**0.8236**	0.4202	0.5565
SVM (*c*: 32 *g*: 0.1)	0.6901	0.7054	**0.6977**
SVM (*c*: 32 *g*: 0.01)	0.6152	**0.7789**	0.6874
CNNdel	0.6894	0.7069	**0.698**

“*c*” stands for penalty factor. “*g*” stands for *σ* in RBF.
